# Sinonasal immunoglobulin G4-related disease: a case report of an atypical and rare entity

**DOI:** 10.1186/s13256-024-04594-0

**Published:** 2024-06-04

**Authors:** Faiq I. Gorial, Nabaa Ihsan Awadh, Shahlaa B. Ali, Sazan Abdulwahab Mirza, Murtadha Hussein Abbas

**Affiliations:** 1 Department of Internal Medicine, Rheumatology Unit, College of Medicine, University of Baghdad, Baghdad, Iraq; 2 Department of Internal Medicine, Rheumatology Unit, Baghdad Teaching Hospital, Baghdad Medical City, Baghdad, Iraq; 3 Department of Internal Medicine, Rheumatology Unit, Al-Imamain Al-Kadhimain Medical City, Alkarkh Health Directorate, Baghdad, Iraq; 4 Department of Pathology and Forensic Medicine, College of Medicine, University of Baghdad, Baghdad, Iraq

**Keywords:** Immunoglobulin G4-related disease, Sinonasal immunoglobulin G4-related disease, Epistaxis, Chronic rhinosinusitis

## Abstract

**Background:**

Immunoglobulin G4-related disease is marked by extensive inflammation and fibrosis of an unknown autoimmune component, with an overall incidence ranging from 0.78 to 1.39 per 10^5^ person-years. Sinonasal immunoglobulin G4-related disease is atypical and exceedingly uncommon in the existing literature, frequently manifesting clinically as chronic rhinosinusitis, epistaxis, and facial pain.

**Case presentation:**

This report describes a 25-year-old Iraqi female who has been suffering from symptoms of chronic rhinosinusitis for 8 years. Despite undergoing several surgeries, there has been no improvement in her symptoms. A tissue biopsy that revealed dense lymphoplasmocytosis with noticeable plasma cell infiltration, storiform fibrosis, and obliterative angitis, along with positive immunohistochemical staining for Immunoglobulin G4 plasma cells, finally confirmed the diagnosis of sinonasal immunoglobulin G4-related disease. The patient responded well to oral prednisolone and methotrexate treatments.

**Conclusions:**

The main objective of the current report is to raise awareness among physicians about the significance of promptly identifying and diagnosing this rarity, thus preventing the adverse consequences linked to delayed diagnosis and treatment initiation.

## Background

Immunoglobulin G4-related disease (IgG4-RD) is a condition characterized by widespread inflammation and fibrosis throughout the body with an autoimmune component of unknown cause. Estimates for the overall incidence of IgG4-RD range from 0.78 to 1.39 per 10^5^ person-years [1]. It is marked by the formation of pseudotumors with storiform fibrosis, obliterative phlebitis, and infiltration of lymphoplasmacytic cells, primarily IgG4^+^ plasma cells. Additionally, individuals with this condition exhibit elevated levels of serum IgG4 [2]. Various organs, including the lacrimal, salivary, and thyroid glands, pancreas, biliary tract, and retroperitoneum, can be affected [3]. Conversely, the occurrence of IgG4-RD as a solitary sinonasal lesion is exceedingly uncommon in existing literature [4–6]. Clinical manifestations of sinonasal IgG4-RD often include facial discomfort, nosebleeds, and persistent rhinosinusitis [7].

This report highlights the first documented case from Iraq, where sinonasal symptoms were the initial sign of IgG4-RD. The purpose is to increase awareness among physicians, especially otolaryngologists, regarding the importance of early recognition and diagnosis, thereby preventing the negative outcomes associated with delayed diagnosis and treatment initiation.

## Case presentation

A 25-year-old Iraqi female presented to the rheumatology consultant clinic in Baghdad Teaching Hospital after experiencing unilateral right-side painless facial swelling, specifically located below the eye on the lateral nasal side, for 1 year. However, the patient’s medical history dates back to 2015, when she started having sporadic episodes of right-side nasal epistaxis brought on by coughing, sneezing, or straining. They progressed from once a month to once a week, so she sought medical attention, and she was advised to undergo local cautery twice on separate occasions.

Regrettably, her disease advanced to impact the left nostril, resulting in the development of crustations and secretions accompanied by post-nasal drip, leading to frequent nighttime coughing. In addition, the patient experienced a symptom of nasal obstruction on the right side. Consequently, in 2017, her physician conducted a surgical procedure to address this issue. The intervention involved correcting the deviation of the nasal septum and releasing adhesions. Nevertheless, her condition progressively deteriorated, and a noticeable nose abnormality became evident, prompting her to have another surgery in 2020 for nasal bone deformity correction with pelvic grafts and adhesion release, which alleviated her complaints to some extent.

Subsequently, early in 2022, the patient experienced significant painless right-side periorbital swelling, increased tears, and no eye redness. It was diagnosed as lacrimal duct obstruction and treated surgically with a favorable outcome. Later, around the end of 2022, she experienced unilateral right-side facial swelling below the eye and lateral to the nose. It was painless and not associated with headaches or visual problems; lastly, a nasal mucosa biopsy was performed, and she was referred to our rheumatology clinic for further evaluation.

Notably, there was no fever, night sweating, loss of appetite, Raynaud’s phenomenon, photosensitivity, oral or genital ulceration, skin tightening, arthralgia, arthritis, morning stiffness, steatorrhea, diarrhea, hematamesis, malena, or abdominal pain throughout this entire period. Reviewing her medical history revealed the patient’s diagnosis of polycystic ovarian syndrome, her management with oral contraceptive pills, and her active participation in a weight loss programme that resulted in a deliberate reduction of 20 kg over an 18-month period. She also has a history of migraines, previously treated with NSAIDs, triptans, and botulinum toxin injections until their cessation in 2020. The patient received prescriptions for topical antibiotics, emollients, and oral antihistamines prior to diagnosis.

The patient’s uneventful pregnancy led to the birth of a healthy daughter with no previous miscarriages or fetal losses. Her lifestyle habits are notable for the absence of significant dental interventions, smoking, or alcohol consumption. Furthermore, there is no familial history of the patient’s condition or autoimmune diseases.

The patient, who is currently unemployed, pursues higher education and is a university candidate. She resides in an urban locale with her family, including a vaccinated indoor cat, ensuring an environment conducive to health and well-being.

The physical examination revealed normal vital signs, including a pulse rate of 90 beats per minute with good volume and regularity, a blood pressure of 110/70 mmHg, a respiratory rate of 16 breaths per minute, a temperature of 36.7 °C, and an oxygen saturation of 98% on room air. There was a mild swelling of the right side of the face below the eye lateral to the nose that was not tender without skin color change. In the submandibular region, there were palpable lymph nodes that were slightly tender, less than 1 cm mobile, and not fixed to underlying structures or skin. Organomegaly, ascites, heart abnormalities, or pulmonary abnormalities were not evident during the systemic examination. Neurological testing confirmed that all cranial nerves were intact. The patient also exhibited normal motor and sensory examinations with no evidence of arthritis.

**Fig. 1:**
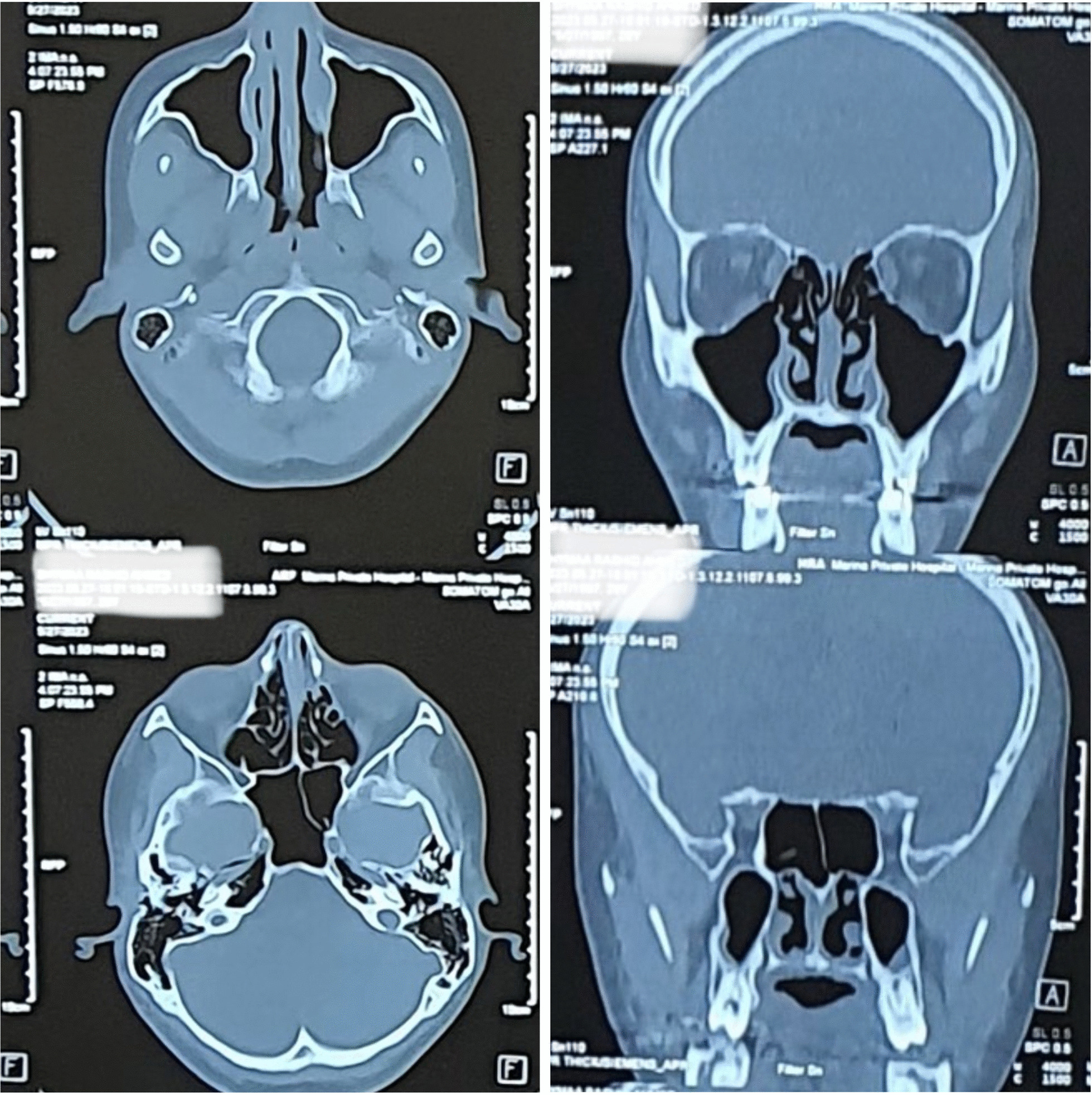
Computed tomography scan of the paranasal sinuses

**Fig. 2:**
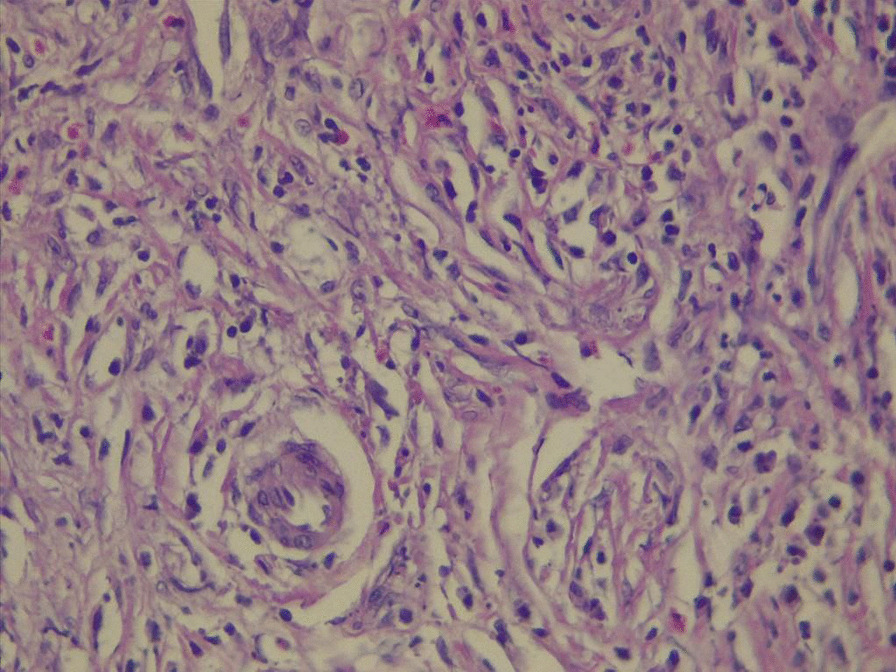
A high-power Hematoxylin and Eosin (H+E) biopsy of the nasal cavity shows dense lymphoplasmocytosis with prominent plasma cell infiltration, storiform fibrosis, and inflammation around small blood vessels

**Fig. 3:**
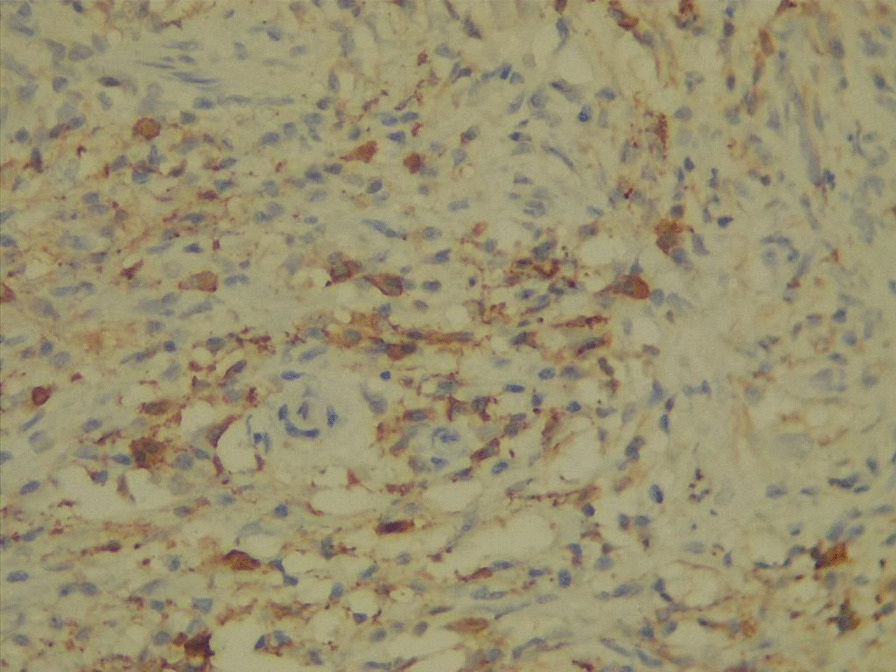
Immunohistochemical stain demonstrating IgG4 positivity

According to the laboratory tests, the erythrocyte sedimentation rate was 34 mm/h, the hemoglobin level was 11 g/dL (11–16), and the ferritin level was 32 ng/mL (20–250). The results of the renal, liver, thyroid, virology, and urinalysis tests were within normal parameters. The results of autoimmune serology tests, including anti-nuclear antibodies, anti-double-stranded DNA antibodies, anti-Ro/SSA antibodies, anti-La/SSB antibodies, and anti-neutrophil cytoplasmic antibodies (ANCA), including cytoplasmic-ANCA (c-ANCA) and perinuclear-ANCA (p-ANCA), were all found to be negative. The serum lgG4 level was 99 mg/dL (2.4–121 mg/dL); refer to Table 1. Neck ultrasounds revealed normal thyroid lobes with homogeneous texture and vascularity, with mixed nodules, mostly solid isoechoic with no calcification, one in the left lobe measuring 2 × 5 mm and another in the right lobe measuring 3 × 4 mm. Bilateral cervical lymph node with intact hilum, with one lymph node in the left submadibular region lobulated in outline measuring 16 × 7 mm and another one near the left carotid vessels measuring 13 × 5.7 mm with an indistinct hilum. The chest radiograph exhibited no abnormalities. The CT scan of the paranasal sinuses (native study) reveals slight thickening of the mucosal lining in the maxillary and right frontal, right ethmoidal sinuses, as well as the nasal cavity (Fig. 1). A nasal mucosa biopsy revealed dense lymphoplasmocytosis with prominent plasma cell infiltration, storiform fibrosis, and obliterative angiitis (Fig. 2). No granuloma and no malignancy were found. An immunohistochemical stain showed increased positivity in plasma cells with IgG4-to-IgG positive plasma cells of more than 40%, as shown in Fig. 3.

**Table 1:** Laboratory parameters of the patient

Laboratory parameter	Results	Normal values
White blood cell count	6.8	4–10 × 10^9^/L
Lymphocyte count	2.2	0.8–2 × 10^9^/L
Granulocyte count	4.2	2–7 × 10^9^/L
Hemoglobin level	11	11–16 g/dl
Mean cell volume (MCV)	78.5	80–100 fL
Platelet count	279	150–400 10^9^/L
ESR	34	0–20 mm/1 h
Total bilirubin	0.65	0.3–1.2 mg/dL
Aspartate transferase (AST)	14.7	Up to 31 U/L
Alanine transaminase (ALT)	15.5	Up to 35 U/L
Alkaline phosphatase	76.3	30–120 U/L
Random blood sugar	103	80–120 mg/dL
Blood urea	39	15–45 mg/dL
Creatinine	0.63	0.5–0.9 mg/dL
Uric acid	3.65	2.5–6.6 mg/dL
Ferritin	32.18	20–250 µg/mL
Vitamin D3 level	21.38	10–50 ng/mL
Thyroid-stimulating hormone (TSH)	0.96	0.4–4 uIU/mL
Free triiodothyronine (T3)	4.1	3.6–6.4 pmol/mL
Free thyroxine (T4)	14.7	10–24 pmol/mL
ANA	0.19	Up to 1
Anti ds-DNA	11.2	Up to 25 U/mL
Anti-Ro (SSA)	0.31	Up to 1 IU/mL
Anti-La (SSB)	0.25	Up to 1 IU/mL
p-ANCA	2.1	Up to 5 IU/mL
c-ANCA	3.2	Up to 5 IU/mL
HIV	Negative	Negative
HBs Ag	Negative	Negative
HCV Ab	Negative	Negative
lgG4 level	99	2.4–121 mg/dL

*ESR* erythrocyte sedimentation rate, *ANA* anti-nuclear antibodies, *Anti ds-DNA* anti-double-stranded DNA antibodies, *c-ANCA* cytoplasmic anti-neutrophil cytoplasmic antibodies, *p-ANCA* perinuclear anti-neutrophil cytoplasmic antibodies, *HIV* human immunodeficiency virus, *HBs Ag* hepatitis B surface antigen, *HCV Ab* hepatitis C antibody

The patient received a treatment regimen consisting of oral prednisolone tablets at a daily dose of 20 mg, methotrexate tablets at a weekly dose of 10 mg, and folic acid tablets at a dose of 5 mg, which resulted in a dramatic response in the form of the disappearance of facial swelling, post-nasal drip, epistaxis, and nasal discharge.

## Discussion

This case report delineates the journey of a 25-year-old female with chronic rhinosinusitis symptoms persisting for 8 years despite multiple surgeries. In this case, biopsy findings consistent with IgG4-related disease (IgG4-RD) in the sinonasal region were unique, as such presentations are extremely rare in the literature. The patient’s robust response to oral prednisolone and methotrexate further underscores the effectiveness of this treatment regimen for managing IgG4-RD-associated symptoms. Furthermore, this report emphasizes the importance of early recognition and diagnosis of sinonasal IgG4-RD to avoid potential adverse outcomes associated with delayed intervention. By documenting this unusual presentation, this case contributes to the growing body of literature on IgG4-RD, emphasizing the necessity for heightened awareness among healthcare providers, particularly otolaryngologists, to facilitate prompt diagnosis and appropriate management of this condition.

IgG4-related disease was described by Kamisawa in the early 2000s [8] as a systemic disease characterized by extensive IgG4-positive plasma cells and T-lymphocyte infiltration that generally (but not always) presents as a mass-like lesion with an elevated serum IgG4 level [9]. Multiple organ systems are involved with IgG4-RD, synchronously or metachronously, and sometimes it may present with isolated organ involvement [10]. After the pancreatic and biliary systems, the head and neck are the next common sites for involvement by the disease [11]. However, sinonasal involvement is rare, with the first case not being described until 2009 by Ishida et al. [12], which is mainly because there is no definitive classification diagnostic criterion for IgG4-related rhinitis or IgG4-related rhinosinusitis established; rather, a three-tiered diagnostic algorithm in a concurrence statement was presented during the first international symposium on IgG4-RD in 2011 [13]. In spite of the fact that most of the patients outlined in literature come from the USA or Japan [14], interestingly, a recent cross-sectional study that collected cases of IgG4-RD head and neck involvement among patients from Europe, America, and Asia delineated a predilection for female patients and patients of Asian descent [3]. Our patient happens to be of Asian origin. So, it would be interesting to look for the reasons that put certain populations at risk, or whether there is a high index of clinical suspicion in certain parts of the world compared with other regions.

Presenting symptoms in cases of sinonasal involvement mimicking rhinitis or rhinosinusitis, possibly delaying or missing investigation for the disease, include chronic rhinitis, epistaxis, nasal obstruction, and facial swelling, as was noted in our case [10]. Although radiographic imaging is useful to determine the extent of disease, sinonasal involvement in IgG4-RD has nonspecific imaging manifestations [15]. The computed tomography (CT) findings of our patient were nondescriptive of the cause of the underlying disease. Impressions implied infection, malignancy, or other autoimmune processes, but no diagnosis could be made.

Many patients may also express elevated serum IgG4. This has been controversial as a diagnostic approach, in which serum levels are normal in 40% of patients with biopsy-proven disease [16]. This statement declares the importance of obtaining a biopsy specimen to confirm the diagnosis of IgG4-RD, depending on which histopathology features are met, including “dense lymphoplasmacytic infiltration with increased IgG4-positive plasmacytosis, fibrosis, often storiform in character, and obliterative phlebitis” [2], so our patient falls under the category of histologically significant [17].

Steroid monotherapy is the first-line treatment for most symptomatic IgG4-RD patients. In addition to steroid use, immunosuppressive drugs (e.g., methotrexate and azathioprine), biologic agents (e.g., rituximab), and surgery are other modalities for the treatment of IgG4-RD. It has been stated in several reports that the use of a combination of steroid and the agents mentioned above had significant responses beyond those of steroid monotherapy, as was observed in our patient [18]. Additionally, two and five cases of IgG4-RD with sinonasal involvement were reported by Keiko Ohno et al. and Masanobu Ueno et al., respectively, for which oral steroid treatment had also shown effective responses [19, 20].

## Conclusion

Sinonasal IgG4-RD is rare with minimal clinical recognition. The likelihood of sinonasal involvement in IgG4-RD should be considered in patients with diffuse swelling of the nasal mucosa or in some cases of refractory sinusitis. Ruling out malignancy, lymphoma, and infection is critical before considering IgG4-RD. Serological tests for IgG4 can be applied as an initial screening test, but a definitive diagnosis is based on histopathological examination. In addition, the diagnosis and treatment of this disease depend on careful analysis of clinical features and effective communication between clinicians and pathologists. Early diagnosis and successful intervention are also essential to controlling and treating the disease. Finally, we assume that this case report will contribute to better recognition of IgG4-RD among physicians and researchers and also focus attention on this rare manifestation of IgG4-RD in the sinonasal cavity.

## Data Availability

Not applicable.

## Abbreviations

IgG4-RD: Immunoglobulin G4-related disease
ANCA: Anti-neutrophil cytoplasmic antibodies
c-ANCA: Cytoplasmic anti-neutrophil cytoplasmic antibodies
p-ANCA: Perinuclear anti-neutrophil cytoplasmic antibodies
NSAIDs: Nonsteroidal antiinflammatory drugs
